# Comparison of physician-certified verbal autopsy with computer-coded verbal autopsy for cause of death assignment in hospitalized patients in low- and middle-income countries: systematic review

**DOI:** 10.1186/1741-7015-12-22

**Published:** 2014-02-04

**Authors:** Jordana Leitao, Nikita Desai, Lukasz Aleksandrowicz, Peter Byass, Pierre Miasnikof, Stephen Tollman, Dewan Alam, Ying Lu, Suresh Kumar Rathi, Abhishek Singh, Wilson Suraweera, Faujdar Ram, Prabhat Jha

**Affiliations:** 1 Centre for Global Heath Research, St Michael’s Hospital, Dalla Lana School of Public Health, University of Toronto, Toronto, Ontario, Canada; 2 Umeå Centre for Global Health Research, Division of Epidemiology and Global Health, Department of Public Health and Clinical Medicine, Umeå University, Umeå, Sweden; 3 Medical Research Council/Wits University Rural Public Health and Health Transitions Research Unit (Agincourt), School of Public Health, Faculty of Health Sciences, University of the Witwatersrand, Johannesburg, South Africa; 4 International Network for the Demographic Evaluation of Populations and Their Health (INDEPTH) Network, Accra, Ghana; 5 International Centre for Diarrhoeal Diseases Research, Bangladesh (ICDDR,B), Dhaka, Bangladesh; 6 Department of Humanities and Social Sciences in the Professions, Steinhardt School of Culture, Education and Human Development, New York University, New York, USA; 7 International Institute for Population Sciences, Mumbai, India

**Keywords:** Causes of death, Computer-coded verbal autopsy, InterVA, King and Lu, Physician-certified verbal autopsy, Random forest, Simplified symptom pattern, Tariff, Validity, Verbal autopsy

## Abstract

**Background:**

Computer-coded verbal autopsy (CCVA) methods to assign causes of death (CODs) for medically unattended deaths have been proposed as an alternative to physician-certified verbal autopsy (PCVA). We conducted a systematic review of 19 published comparison studies (from 684 evaluated), most of which used hospital-based deaths as the reference standard. We assessed the performance of PCVA and five CCVA methods: Random Forest, Tariff, InterVA, King-Lu, and Simplified Symptom Pattern.

**Methods:**

The reviewed studies assessed methods’ performance through various metrics: sensitivity, specificity, and chance-corrected concordance for coding individual deaths, and cause-specific mortality fraction (CSMF) error and CSMF accuracy at the population level. These results were summarized into means, medians, and ranges.

**Results:**

The 19 studies ranged from 200 to 50,000 deaths per study (total over 116,000 deaths). Sensitivity of PCVA versus hospital-assigned COD varied widely by cause, but showed consistently high specificity. PCVA and CCVA methods had an overall chance-corrected concordance of about 50% or lower, across all ages and CODs. At the population level, the relative CSMF error between PCVA and hospital-based deaths indicated good performance for most CODs. Random Forest had the best CSMF accuracy performance, followed closely by PCVA and the other CCVA methods, but with lower values for InterVA-3.

**Conclusions:**

There is no single best-performing coding method for verbal autopsies across various studies and metrics. There is little current justification for CCVA to replace PCVA, particularly as physician diagnosis remains the worldwide standard for clinical diagnosis on live patients. Further assessments and large accessible datasets on which to train and test combinations of methods are required, particularly for rural deaths without medical attention.

## Background

Most of the 48 million deaths that occurred in 2010 in low- and middle-income countries (LMICs) occurred without medical attention, in homes in rural areas [1-3]. Verbal autopsy (VA) has been increasingly used in LMICs to define causes of death (CODs). VA entails an interview with a relative or close associate of the deceased, using a questionnaire to elicit information on the signs, symptoms and chronological sequence of events during the final illness leading to death. VA questionnaires vary, but generally comprise a mix of closed questions and open or semi-structured narratives. COD surveys have mostly informed specific research needs in small populations, and have largely focused on child or maternal deaths [4]. Increasingly there is interest in the use of VA for large-scale nationally representative COD surveys, such as the ongoing Indian Million Death Study (MDS) [5,6] and others in Africa [7].

**Figure 1:**
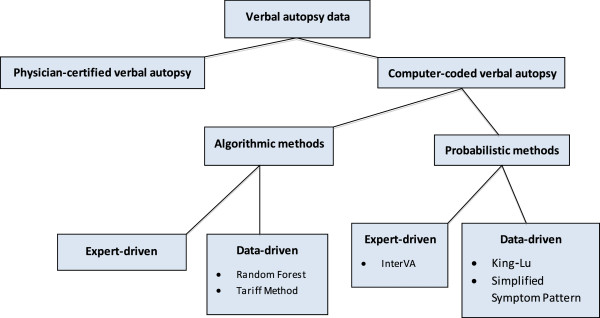
Classification of verbal autopsy interpretation methods.

Methods to assign COD in VAs can be categorized as physician-certified verbal autopsy (PCVA) or computer-coded verbal autopsy (CCVA) (Figure 1). PCVA typically involves at least two physicians examining each record, with adjudication done by a consensus review or by a third physician [8,9]. In recent years, there has been interest in using CCVA to improve inter-observer agreement, consistency and comparability, and to make the coding of VAs faster and cheaper. We conducted a systematic review of studies assessing the performance of CCVA and PCVA methods. Most studies used hospital-based diagnosis as the reference comparison. Thus, we also discuss the relevance of the findings to rural or medically unattended deaths, populations among whom VA studies are needed most urgently.

## Methods

We conducted a systematic review of VA performance studies, adhering broadly to PRISMA guidelines [10], and compared five CCVA methods to PCVA: two data-driven algorithms, Random Forest (RF) and Tariff; InterVA, an expert-based probabilistic method; and two data-driven probabilistic methods, King-Lu (KL) and Simplified Symptom Pattern (SSP) (Figure 1) [11-16]. Additional file 1 offers background information on these methods. Various versions of InterVA models have been available in the public domain since 2003; most of the studies here used InterVA-3 rather than the current InterVA-4 model [15,17].

Two of the authors (JL, ND) independently searched three online databases (PubMed, Popline, and LILACS) for relevant studies; disagreements were handled by JL, and a senior author (PJ) resolved any differences. A search of the EMBASE database yielded no additional relevant studies. Key terms employed in the electronic searches were verbal autopsy, cause of death, validity, validation, performance, accuracy, and assessment. The literature search was concluded in June 2013.

The validity of VA is dependent on its many components and there is a high degree of variability between studies in terms of field procedures, questionnaires used, CODs assessed, recall by respondents, and metrics of performance, among others. To ensure comparability and quality of studies, we included only studies that fitted our eligibility criteria. Firstly, as the validity of VA depends heavily on the questions used, only studies using the most common and validated questionnaires were eligible. These included an adaptation or sub-version of the following VA questionnaires: World Health Organization VA tools; INDEPTH; London School of Hygiene and Tropical Medicine VA; Sample Vital Registration with Verbal Autopsy; Routine, Reliable, Representative and Resampled Household Investigation of Mortality with Medical Evaluation (MDS); or questionnaires used in the mortality surveillance systems of Tanzania and China [5,18-25]. Guidance for these questionnaires also came from a World Health Organization review meeting on formulation of standard guidelines for its VA tool [26]. Secondly, PCVA coding must have been specifically carried out by physicians and not by other types of health professionals. Lastly, the study had to include at least 100 deaths for studies examining a single COD, and at least 1,000 total deaths for studies assessing various CODs.

**Figure 2:**
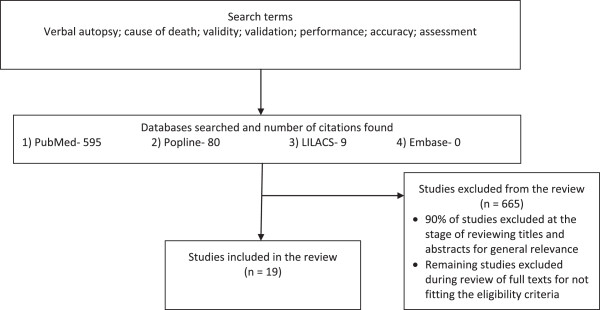
**Systematic review process of studies assessing the performance of physician-certified verbal autopsy and computer-coded verbal autopsy methods.** Search terms used: verbal autopsy, cause of death, validity, validation, performance, accuracy, assessment.

The most important underlying measure of quality in each study was the accuracy of diagnosis of the reference standard, though this could not be addressed through any additional criteria in this review. The search imposed no restriction on the period of publication or language used, and resulted in the selection of 19 studies from a total of 684 screened articles. The systematic review process is illustrated in Figure 2.

Two of the authors independently extracted the relevant data from the selected studies. Various metrics are used to assess the performance of VA methods. We selected the most commonly reported metrics *a priori* so as to increase comparability across the studies: sensitivity, specificity, and the cause-specific mortality fraction (CSMF) error (the relative difference between the VA and the reference standard CSMFs). The reference diagnosis in most studies was medically-assigned COD from hospital-based deaths (Additional file 2). While there is no international consensus on benchmark values of validity, a working rule of thumb is to seek a sensitivity and specificity of at least 80% at the individual level, and a minimum sensitivity of 50% and specificity of 90% at the population level. Low individual agreement may still produce accurate CSMFs at the population level as long as false positives and false negatives balance each other out. Hence, sensitivity thresholds are set lower than those for specificity [26,27]. CSMFs were determined as the proportion of all deaths that were attributable to a specific COD. In studies where CSMF error was not reported, we calculated the relative difference between CSMFs from VA and the reference standard, for selected CODs. While there is also no agreed benchmark value for CSMF error, we considered a relative difference of at least 10% between CSMFs to represent significant disagreement.

The RF, Tariff, and SSP methods have only been tested by the Institute of Health Metrics and Evaluation (IHME) [21], and at the time of writing of this manuscript, the datasets and methods for these hospital-based comparisons were not in the public domain. From these studies, we report the chance-corrected concordance as a measure of individual performance, and CSMF accuracy as a measure of population-level performance. IHME assessed the performance of VA methods with the inclusion and exclusion of free text from the narrative and household recall of healthcare experience. We chose to only use the results for which performance was assessed with the inclusion of all constituent parts of a VA questionnaire, as this is the form in which VA is administered conventionally. InterVA-3 was the only method for which IHME did not report performance for specific causes with the inclusion of free text and household recall of healthcare experience. To ensure a fair comparison across the methods, we did not include the findings for InterVA-3 for chance-corrected concordance or CSMF accuracy by cause. Estimates of performance for adults, children, neonates, and all ages combined from the IHME group of studies were reported, while only CODs for all ages combined were available in the remainder of studies. Given the large amount of heterogeneity in the studies, including variation in methods of data collection, forms used, age groups studied, and single versus double coding by physicians, we did not attempt formal meta-analytic summary measures such as quantification of measures of heterogeneity. Rather, simple means or medians, and ranges were calculated across the various comparison studies.

## Results

The review identified 19 eligible studies conducted between 1992 and 2012 assessing the performance of VA methods [11-16,28-40]. Additional file 2 summarizes the main characteristics of the included studies. The size of the study samples ranged from 200 to 50,000 deaths, for a total of 116,679 deaths. Fifteen out of nineteen studies used hospital-assigned COD from medical records as the reference standard, while the remaining four assessed InterVA-3 using PCVA as a reference standard. Fifteen studies assessed performance across a range of CODs, and four assessed a single COD. Eleven studies assessed performance across all ages, while seven assessed performance specifically in adolescents and adults (defined as age 12 years and above), and one in children under 5 years of age. We included eight studies evaluating the performance of PCVA, seven studies evaluating InterVA, and one study for each of the KL, RF, Tariff and SSP methods.

### Individual-level cause of death assignment

Table 1 shows the means and ranges of sensitivity and specificity reported for PCVA for 21 major CODs. Sensitivity varied considerably, with wide ranges of estimates across specific CODs (0% to 98%). On average, PCVA was reasonably accurate when compared to hospital-based diagnosis for HIV/AIDS, site-specific cancers, cirrhosis of the liver, stroke, chronic respiratory diseases, maternal deaths, road traffic accidents, and other injuries. PCVA achieved the highest levels of accuracy in certifying road traffic accidents and digestive cancers with median sensitivity values of 97% (97% to 98%) and 84% (80% to 89%), respectively. By contrast, PCVA was relatively poor at confirming hospital-based diagnosis of infections, other digestive diseases, nutritional conditions, heart diseases, renal and other endocrine diseases, and neonatal conditions. PCVA had the poorest performance for renal and other endocrine diseases, with a mean sensitivity of 32% (13% to 54%). PCVA yielded good levels of specificity of at least 90% for the majority of CODs, with the exception of malaria, with a mean of 89% (0% to 100%). In one hospital-based study, InterVA-3 appears to more accurately ascertain HIV/AIDS than PCVA, with a mean sensitivity of 87%, but with a lower specificity of 77% (76% to 78%; data not shown). Another study found InterVA-3 to have a sensitivity of 82% and specificity of 78% in the certification of tuberculosis in relation to PCVA [39,40].

**Table 1:** Mean, ranges and number of reviewed studies for sensitivity and specificity estimates of physician-certified verbal autopsy for selected causes of death, among hospital-based deaths

	**Sensitivity**			**Specificity**		
	**Mean (%)**	**Range**	**Number of studies**	**Mean (%)**	**Range**	**Number of studies**
**Infections and parasitic diseases and maternal deaths**						
Tuberculosis	39	18 to 62	3	97	93 to 99	2
HIV/AIDS	59^a^	0 to 61	3	90^a^	0 to 96	3
Diarrheal diseases^b^	38^a^	0 to 75	2	96	94 to 99	2
Malaria^b^	60^a^	0 to 67	2	89^a^	0 to 100	2
Pneumonia	42	18 to 75	4	93	84 to 99	3
Maternal deaths	63	-	1	100	-	1
**Neonatal conditions**						
Prematurity or low birth weight	48	-	1	95	-	1
Birth asphyxia or birth trauma	43	-	1	90	-	1
Neonatal infections	31	11 to 50	2	100	99 to 100	2
**Noncommunicable diseases**						
Nutritional conditions	33^a^	0 to 58	2	94	87 to 99	2
Digestive cancers	82^a^	56 to 96	3	100	99 to 100	2
Respiratory cancers	84	80 to 89	2	99	-	1
Other cancers	61^a^	27 to 95	5	99	98 to 99	2
Heart disease	39	16 to 64	3	98	98 to 99	2
Stroke	71	63 to 82	3	95	94 to 97	2
Chronic respiratory diseases	61	60 to 62	2	98	-	1
Cirrhosis of the liver	58	45 to 71	2	98	-	1
Other digestive diseases	36	21 to 52	2	99	-	1
Renal or endocrine diseases	32	13 to 54	3	99	99 to 99.4	2
**Injuries**						
Road traffic accidents	97	97 to 98	2	100	-	1
Other injuries	57	35.3 to 74	3	100	99 to 100	2

^a^Median was used instead of means due to outlier values in the ranges of estimates. However, the medians and means yielded similar results (that is, the pooled studies gave PCVA a sensitivity mean of 80.1% and a median of 81.9% for ascertaining digestive cancers). ^b^Although two studies were used to generate results for these CODs, the studies provided results for several population sub-samples.

Table 2 presents the median chance-corrected concordance from the IHME group of hospital-based studies for five VA methods, by age. All the VA methods had an overall chance-corrected concordance lower than 50% for combined age groups. RF reported the highest chance-corrected concordance (45%), followed closely by PCVA (42%) and SSP (40%). Within age groups, RF and SSP achieved moderate levels of performance in children (51% and 52%, respectively). Median values of chance-corrected concordance were calculated for selected CODs (Additional file 3), with PCVA, Tariff, RF and SSP trading best performance by individual CODs; all methods had a chance-corrected concordance above 50% for HIV/AIDS (54% to 64%), maternal deaths (64% to 89%), stroke (50% to 63%), road traffic accidents (66% to 85%) and other injuries (57% to 61%). The highest accuracy was achieved for road traffic accidents (85%, by RF and PCVA) and maternal deaths (89% and 75%, by SSP and RF). Largely, all the methods performed poorly in certifying various infections, particularly pneumonia (17% to 27%) and other infections (5% to 25%). Among noncommunicable causes, similarly low performance was seen for vascular diseases (9% to 30%), other digestive diseases (21% to 27%), chronic respiratory diseases (43% to 49%), renal and other endocrine diseases (12% to 33%), and neonatal conditions (6% to 48%).

**Table 2:** Median chance-corrected concordance (%) by age, for all causes of death, for physician-certified verbal autopsy, InterVA-3, Tariff, Random Forest and Simplified Symptom Pattern, among hospital-based deaths

			**IHME sub-studies**		
	**Physician-certified verbal autopsy**	**InterVA-3**	**Tariff**	**Random forest**	**Simplified symptom pattern**
**Adults**	45	25	45	48	46
**Children**	48	25	39	51	52
**Neonates**	33	7	24	35	33
**All ages**	42	19	36	45	43

The IHME studies provide an uncertainty limit, but these do not seem to reflect the true underlying source of error in the estimates, and to avoid false precision, we do not show these [41]. IHME: Institute for Health Metrics and Evaluation.

### Population-level cause of death assignment

**Figure 3:**
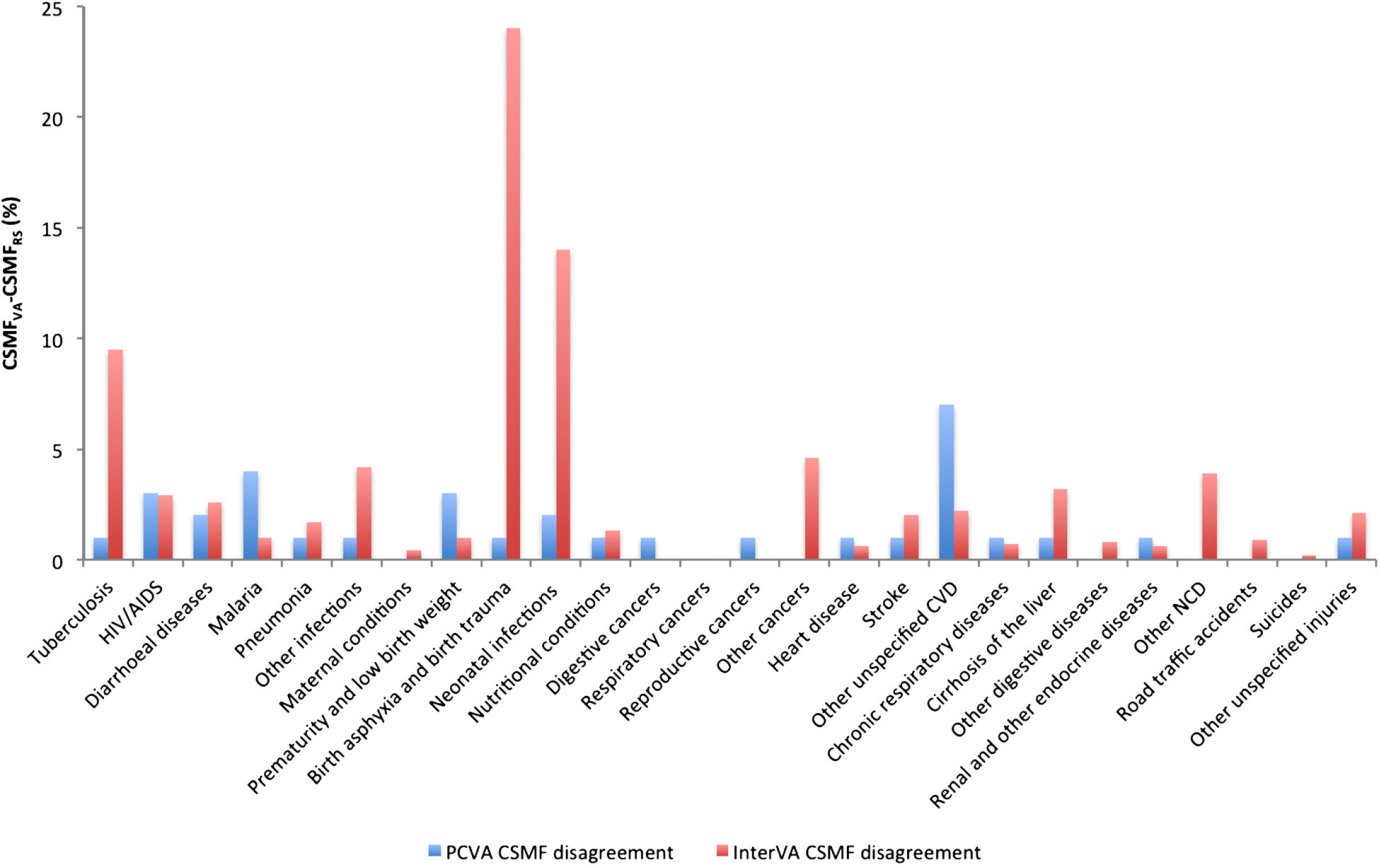
**Cause-specific mortality fraction relative error between physician-certified verbal autopsy and InterVA versus reference standards, by cause of death.** CSMF error is presented between PCVA and hospital-based deaths, and InterVA-3 and PCVA, from reviewed studies. The bars of the graph are not comparable between PCVA and InterVA-3, as each used a different reference standard. CSMF, cause-specific mortality fraction; PCVA, physician-certified verbal autopsy.

The CSMF error between PCVA and hospital-based deaths, and between InterVA-3 and PCVA, are shown in Figure 3. The CSMFs for nearly all causes estimated by PCVA did not differ significantly from the reference standard. The notable exception was other cardiovascular diseases, with a mean difference of 7%, ranging between 4% and 10%. InterVA-3 had close agreement in CSMF estimation compared with PCVA for most of the selected CODs. However, InterVA-3 had considerably higher CSMF relative errors for tuberculosis (10%), birth asphyxia and birth trauma (24%), and neonatal infections (14%).

The median CSMF accuracy from IHME hospital-based studies for adults, children, neonates and all ages combined, for five VA methods, is shown in Table 3. At all ages combined, RF had the highest median CSMF accuracy (0.77), followed by SSP (0.74), tariff (0.71), KL (0.70), PCVA (0.68), and InterVA-3 (0.52). Within age groups, performance between the methods followed similar trends as above, though KL achieved the best performance (0.8) for neonates. However, the results from the IHME studies were based on data-driven models that were built from the same dataset that was used to evaluate their performance [42]. Consequently, the results for RF, SSP and Tariff represented measures of internal validity, alongside the IHME studies of PCVA and InterVA-3, which reported measures of external validity against the IHME dataset.

**Table 3:** Median cause-specific mortality fraction accuracy by age for all causes of death, among hospital-based deaths

				**IHME sub-studies**		
	**Physician-certified verbal autopsy**	**InterVA-3**	**King and Lu**	**Tariff**	**Random forest**	**Simplified symptom pattern**
**Adults**	0.68	0.55	0.67	0.75	0.77	0.71
**Children**	0.68	0.52	0.70	0.71	0.78	0.74
**Neonates**	0.73	0.41	0.80	0.68	0.73	0.75
**All ages**	0.68	0.52	0.70	0.71	0.77	0.74

## Discussion

Our systematic review finds that no single VA method consistently outperformed the others across selected CODs, for both individual- and population-level COD assignment. One challenging aspect of comparing validation studies is the variation in study design, particularly in regards to reference standards and performance measures used. In hospital-based comparison studies, each PCVA and CCVA method had unique performance strengths for various CODs. This is expected, as probabilistic methods such as KL, InterVA and SSP assign a fixed probability between each symptom indicator and each cause (for example, the probability of loose bowel movements being associated with death from diarrheal disease), though in reality, for any given COD, symptomatology might well differ between individuals. Moreover, in comparison to PCVA, CCVA is weak at establishing the chronology of events, which may have consequences for diagnosis. For example, a history of cough or fever followed by chest pain is more likely to indicate pneumonia than a history of chest pain followed by a cough or fever, which may signal cardiac conditions [43]. Moreover, physicians’ perceptions of local epidemiology can influence their diagnosis in the absence of clear etiology, introducing bias. This could be the case in the slight excess coding of fever deaths such as malaria (and under-coding of fevers of unknown origin) in areas of India where malaria remains common [6]. Finally, the current clinical standard for diagnosis in routine medical care worldwide remains a physician interview, and it is hard to imagine any patient accepting a computer-based diagnosis without physician scrutiny.

One frequently stated advantage of CCVA methods over PCVA is their repeatability and the temporal and spatial comparability of CSMF estimation. This is likely true, though a small, independent resample of the MDS showed broad agreement in physician coding with the original CODs assigned. Differences between physicians’ assignment of COD exist at the individual level, but these differences appear to have little impact on CSMF estimation, given that misclassification tends to be bidirectional [44].

The development of data-driven algorithms requires training and test datasets. Typically, a VA dataset containing information about signs and symptoms coupled with assigned CODs is used to train algorithms that then assign CODs to a test dataset [45-48]. Data-derived methods, especially those trained on hospital deaths, may be limited in three ways. First, development and testing on the same dataset may be self-reinforcing, in that any bias in the VA survey would be internalized during testing, and hence inflate the reported accuracy, as documented recently in the IHME sub-studies [42]. Second, methods trained on hospital-based causes may not have a sufficiently large sample from which to train on the CODs that are not common in hospital settings such as malaria. Finally, training on hospital-based deaths has uncertain external validity for non-hospital deaths, because the symptomatology (as well as the recall of the deceased’s relatives) may differ between these populations. This review emphasizes that each method has particular advantages for certain CODs, and that the best performance may come from using multiple methods, including the use of natural language processing [49]. This places particular emphasis on the need for expanded datasets for training and testing to further compare CCVA methods with each other. Currently, InterVA is the only CCVA method that determines COD from a universally applicable model, which is not trained on any specific dataset. InterVA thus trades maximization of performance in specific contexts with a reasonable level of generalizability and comparability.

Two other operational aspects need to be considered when designing VA studies. First, as both CCVA and PCVA methods have been shown to generate reasonably robust COD estimates at the population level, the most pressing need is to implement VA surveys much more widely, particularly large-scale nationally representative surveys [1,3,50]. This would be a substantial advancement over the dearth of COD data that exist in most LMICs. Second, PCVA and CCVA have unique strengths as coding methods; while PCVA is more dependent on the quality of fieldwork and record-keeping than CCVA, it is also more transparent, and the adjudication trail from one physician to the next and final code is easily followed. CCVA methods involve a ‘black box’ nature that implies a leap of faith in trusting sometimes complex and inaccessible assumptions. The MDS uses e-health records to enable anonymous electronic coding by 300 physicians, which makes coding faster than traditional paper-based methods. The IHME group of studies found that, generally, the performance of the VA methods improved with the inclusion of free text from the narrative and information from health care use (data not shown), which is similar to findings from the MDS [5]. This suggests that a future strategy is to pair PCVA with CCVA, to assist physicians’ decision making and further improve and standardize physician coding. Currently, the Indian MDS offers all coders a differential diagnosis based on the initial physician disagreements of 130,000 deaths from 2001 to 2003 [44].

Metrics of performance were not consistent across the studies. For InterVA, the main metric available was the agreement between CSMF estimated by InterVA and PCVA, which showed reasonably similar results for most causes. When considering this agreement, its interpretation as a measure of accuracy at the population level must be made bearing in mind that PCVA is not 100% reliable and does not yield high accuracy for all CODs. Although sensitivity values for PCVA varied widely across causes and settings, the specificity was generally high, ranging from 89% to 100%. Specificity is more important than sensitivity when comparing performance to the true underlying CSMFs. Even a small loss of specificity leads to underestimation of CSMF errors [27].

Finally, the most important limitation of the studies is their use of mostly urban-based hospital reference standards. The accompanying paper by Aleksandrowicz *et al*. demonstrates that, in India, there are marked differences in the COD structure between urban or hospital deaths, and rural or medically unattended deaths [44], even after taking into account differences in education or other social status. Relatives who have had little interaction with doctors and nurses during the events preceding death might describe signs and symptoms very differently from those whose relatives died in the hospital, and whose accounts may be biased by what they are told by the doctors. Were India’s COD estimates based solely on hospital data, the CSMF proportions would differ substantially [41,51,52]. The most glaring example is the 13-fold higher estimate of malaria deaths in India based on rural VA, versus hospital-based malaria diagnoses [6].

## Conclusions

PCVA and CCVA methods differ in their performance of coding hospital-based deaths, and there is no single best-performing method. Further testing of CCVAs is required to improve the performance of COD-assignment, and the comparability between methods. In particular, there is a need for large, accessible datasets on which to train and test automated methods in conjunction with PCVA. More importantly, nationally representative VA studies are required to improve the dearth of COD data in most LMICs. These representative studies offer the best hope to extend such testing from the hospital to the community level, so as to compare various VA methods where most deaths actually occur in LMICs - in rural households without medical attention.

## Abbreviations

CCVA: computer-coded verbal autopsy; COD: cause of death; CSMF: cause-specific mortality fraction; IHME: Institute of Health Metrics and Evaluation; KL: King and Lu; LMIC: low- and middle-income countries; MDS: Million Death Study; PCVA: physician-certified verbal autopsy; RF: Random Forest; SSP: Simplified Symptom Pattern; VA: verbal autopsy.

## Competing interests

The authors declare that they have no competing interests.

## Authors' contributions

All authors contributed equally to this work. JL, ND, LA and PJ did the data analyses of published studies. All authors read and approved the final manuscript.

## Supplementary Material

Additional file 1 Description of PCVA and CCVA algorithmic and probabilistic methods. Click here for file

Additional file 2 Summary characteristics of reviewed comparison studies. Click here for file

Additional file 3 Chance-corrected concordance by cause for PCVA, Tariff, RF and SSP. Click here for file
