# Supplementary material for: Comparison of physician-certified verbal autopsy with computer-coded verbal autopsy for cause of death assignment in hospitalized patients in low- and middle-income countries: systematic review
Source: BMC Med. 2014 Feb 4;12:22. doi: 10.1186/1741-7015-12-22 (PMC3912516; doi:10.1186/1741-7015-12-22)
Supplement: Additional file 1 — Description of PCVA and CCVA algorithmic and probabilistic methods. [file 1741-7015-12-22-S1.doc]

### Additional file 1- Description of PCVA and CCVA algorithmic and probabilistic methods

### Physician Certified Verbal Autopsy (PCVA)

Physician review of VA was the first method used to interpret VA data and still is the most widely used. Variations in PCVA methodology exist, such as the number of individual physicians or of a panel of physicians used to derive diagnosis, and the type of physicians used (generalists versus specialized physicians). In most cases, 2 generalist physicians review and code independently the VA data; when there is disagreement in COD assignment between physicians, an additional review is sought to reach a consensus diagnosis. If there is no agreement on the cause, the death is recorded as “indeterminate”. Most studies adopt the steps for VA coding recommended by the WHO, where in cases involving discrepancies between physicians, adjudication is done by a third physician to determine the final COD assignment[1-3].

### Computer-Coded Verbal Autopsy (CCVA) Methods

Concerns over interobserver agreement, consistency and comparability of physicians COD assignment over place and time led to the development of CCVA methods. With CCVA methods, COD are derived using predefined criteria, enabling the automation of coding process. Current CCVA methods for VA data interpretation are mainly split into algorithmic and probabilistic methods, which based on their decision rules can be either data-driven or derived from experts’ encapsulated opinion t[1, 4].

#### Algorithmic Approaches

Expert-derived algorithms can be developed using textbook descriptions, existing algorithms, local experience, or more commonly physician panels, distilling clinical knowledge into standard rules. Alternatively, algorithms can be generated from VA data through the use of standard statistical techniques to identify more discriminating cause-specific symptoms. The development of data-driven algorithms requires training and test datasets. Typically, a VA dataset containing information about signs and symptoms coupled with known mortality data, ideally medically confirmed, is used to train algorithms and then assign COD in the test dataset[5–8]. Two data-driven algorithmic approaches developed in recent years by the Population Health Metric Research Consortium have shown the most promise: the Random Forest (RF) and Tariff methods.

RF is a machine learning method based on ‘decision trees’ that resembles expert-derived algorithms but which are instead generated automatically by a randomized algorithm from resampled VA training data. Starting from the root, a decision tree is constructed sequentially and at each node, the algorithm selects a random subset of signs and symptoms. Based on the labeled examples from training set, the algorithm branches on the one that best distinguishes between causes. The PHMRC method performs ‘pairwise coupling’, training RF to create 100 decision trees to differentiate between each pair of causes. Subsequently, scores for each cause are generated based on the number of trees that predict each COD and are tallied in a Test Score Matrix. To enable comparability of individual COD scores, RF normalizes the scores for each COD by converting the Test Score Matrix into a Test Rank Matrix[9].

The Tariff method is a simpler additive algorithmic method based on the assumption that highly informative signs and symptoms exist for each COD. The method works by calculating a score or “tariff” that reflects how informative each indicator is for each COD. Tariffs are generated from training datasets and are computed as a function of the fraction of deaths for each indicator that has a positive response. For each case, depending on the response pattern from the VA questionnaire, tariffs are summed yielding an indicator-specific tariff score for each possible COD[10].

#### Probabilistic Approaches

Instead of assessing the presence or absence of COD based on positive or negative responses to COD-specific indicators, probabilistic approaches simultaneously quantify the probabilities of various COD. Assigned probabilities are conditional on population-level and on COD-specific probabilities of indicators being reported for a particular cause. Depending on how a priori probabilities are determined, probabilistic approaches can also be thought as either expert-derived or data-driven[1].

InterVA has been the longest standing of the methods and the latest version of the method, InterVA-4, accumulates experience and research findings from past versions and is aligned with the 2012 WHO VA instrument[11]. The method developed by *Byass et al.* applies Bayesian probabilistic modeling to determine up to three possible COD with their associated likelihoods for each case. The Bayes’ theorem associates the probability of a certain COD given the presence of a certain indicator, with the unconditional probability of the same COD and the conditional probability of the indicator given the COD. InterVA has used the clinical insight of expert panels to determine the conditional probabilities of signs, symptoms and circumstances leading to death (indicators) in regards to their affiliation with specific COD. Besides yielding up to three COD with their respective probabilities, InterVA also provides a certainty indicator that can assist in the interpretation of COD assignment. If no single cause has a likelihood of at least 0.4, the model classifies the cause as indeterminate, and multiple causes are only assigned if they have half of the likelihood of the leading COD. Another feature of the method is the modulation of unconditional probabilities for malaria (and by association sickle-cell), HIV/AIDS to reflect their occurrence in particular settings[1, 12–16].

With data-driven probabilistic methods, the probability of a certain indicator appearing for a particular COD is determined using labeled examples from a training dataset[1]. Two such probabilistic data-driven methods are the direct CSMF estimation method by King and Lu (KL), and the Simplified Symptom Pattern (SSP) method by the PHMRC.

The KL method estimates aggregate proportions to directly determine the CSMFs of a dataset, without assigning COD at the individual level. To capture the complex relationships between indicators, the KL method centers on the assumption that sign and symptom profiles of COD in the training and test datasets are similar. Using Bayesian techniques, the method selects indicators from VA instruments that optimize performance, and assign posterior probabilities. In contrast with the other described methods, KL is not dependent of the inclusion in VA questionnaires of indicators with high specificity and sensitivity for specific COD; in addition, it can be trained on any dataset with known COD to directly estimate CSMF in test datasets [5, 17].

The PHMRC developed a simplified and improved version of the Symptom Pattern (SP) method generated by *Murray et al.* in 2007. The original SP was a Bayesian method that combined principles of KL and InterVA methods to ascertain both CSMF estimation and individual COD assignment ^(Fottrell E, 2010)^. The Simplified Symptom Pattern (SSP) method introduced in 2011 differs from the SP in a number of ways: symptom clusters of 10 are used as opposed to symptom clusters of one; no longer uses KL direct CSMF as prior, using instead a uniform prior; and uses all indicators from VA questionnaires. It was also found that SSP is enhanced by generating models for each individual COD, by predicting if a death is from a specific COD compared to all others, and then selecting the cause with the highest posterior probability across each of the specific models[18, 19].

**Bibliography**

1. Fottrell E, Byass P: Verbal Autopsy: Methods in Transition. *Epidemiol Rev* 2010, 32:38–55.

2. RGI-CGHR: *Prospective Study of Million Deaths in India: Technical Document No VIII: Health Care Professional’s Manual for Assigning Causes of Death Based on RHIME Reports*. University of Toronto: RGI-CGHR, University of Toronto; 2011.

3. Yé M, Diboulo E, Niamba L, Sié A, Coulibaly B, Bagagnan C, Dembélé J, Ramroth H: An improved method for physician-certified verbal autopsy reduces the rate of discrepancy: experiences in the Nouna Health and Demographic Surveillance Site (NHDSS), Burkina Faso. *Popul Heal Metrics* 2011, 9:34.

4. Chandramohan D: Validation and validity of verbal autopsy procedures. *Popul Heal Metrics* 2011, 9:22.

5. King G, Lu Y: Verbal Autopsy Methods with Multiple Causes of Death. *Stat Sci* 2008, 23:78–91.

6. Soleman N, Chandramohan D, Shibuya K: Verbal autopsy: current practices and challenges. *Bull World Health Organ* 2006, 84:239–245.

7. Quigley MA, Armstrong Schellenberg JR, Snow RW: Algorithms for verbal autopsies: a validation study in Kenyan children. *Bull World Health Organ* 1996, 74:147–154.

8. Quigley MA, Chandramohan D, Rodrigues LC: Diagnostic Accuracy of Physician Review, Expert Algorithms and Data-Derived Algorithms in Adult Verbal Autopsies. *Int J Epidemiol* 1999, 28:1081–1087.

9. Flaxman AD, Vahdatpour A, Green S, James SL, Murray CJ: Random forests for verbal autopsy analysis: multisite validation study using clinical diagnostic gold standards. *Popul Heal Metrics* 2011, 9:29.

10. James SL, Flaxman AD, Murray CJ, PHMRC: Performance of the Tariff Method: validation of a simple additive algorithm for analysis of verbal autopsies. *Popul Heal Metrics* 2011, 9:31.

11. Byass P, Chandramohan D, Clark SJ, D’Ambruoso L, Fottrell E, Graham WJ, Herbst AJ, Hodgson A, Hounton S, Kahn K, Krishnan A, Leitao J, Odhiambo F, Sankoh OA, Tollman SM: Strengthening standardised interpretation of verbal autopsy data: the new InterVA-4 tool. *Glob Heal Action* 2012, 5: 19281.

12. Bauni E, Ndila C, Mochamah G, Nyutu G, Matata L, Ondieki C, Mambo B, Mutinda M, Tsofa B, Maitha E, Etyang A, Williams TN: Validating physician-certified verbal autopsy and probabilistic modeling (InterVA) approaches to verbal autopsy interpretation using hospital causes of adult deaths. *Popul Heal Metrics* 2011, 9:49.

13. Oti Samuel, Catherine K: *Verbal Autopsy Interpretation: a Comparative Analysis of the InterVA Model Versus Physician Review in Determining Causes of Death in the Nairobi DSS*. *Popul Heal Metrics* 2010, 8:21.

14. Tensou B, Araya T, Telake DS, Byass P, Berhane Y, Kebebew T, Sanders EJ, Reniers G, Tensou B, Araya T, Telake DS, Byass P, Berhane Y, Kebebew T, Sanders EJ, Reniers G: Evaluating the InterVA model for determining AIDS mortality from verbal autopsies in the adult population of Addis Ababa. *Trop Med Int Heal*  2010, 15:547–553.

15. Fantahun M, Fottrell E, Berhane Y, Wall S, Högberg U, Byass P: Assessing a new approach to verbal autopsy interpretation in a rural Ethiopian community: the InterVA model. *Bull World Health Organ* 2006, 84:204–210.

16. Vergnano S, Fottrell E, Osrin D, Kazembe PN, Mwansambo C, Manandhar DS, Munjanja SP, Byass P, Lewycka S, Costello A: Adaptation of a probabilistic method (InterVA) of verbal autopsy to improve the interpretation of cause of stillbirth and neonatal death in Malawi, Nepal, and Zimbabwe. *Popul Heal Metrics* 2011, 9:48.

17. Flaxman AD, Vahdatpour A, James SL, Birnbaum JK, Murray CJ, PHMRC: Direct estimation of cause-specific mortality fractions from verbal autopsies: multisite validation study using clinical diagnostic gold standards. *Popul Heal Metrics* 2011, 9:35.

18. Murray CJ, James SL, Birnbaum JK, Freeman MK, Lozano R, Lopez AD: Simplified Symptom Pattern Method for verbal autopsy analysis: multisite validation study using clinical diagnostic gold standards. *Popul Heal Metrics* 2011, 9:30.

19. Murray CJL, Lopez AD, Feehan DM, Peter ST, Yang G: Validation of the Symptom Pattern Method for Analyzing Verbal Autopsy Data. *PLoS Med* 2007, 4:e327.
