# Supplementary material for: Comparison of physician-certified verbal autopsy with computer-coded verbal autopsy for cause of death assignment in hospitalized patients in low- and middle-income countries: systematic review
Source: BMC Med. 2014 Feb 4;12:22. doi: 10.1186/1741-7015-12-22 (PMC3912516; doi:10.1186/1741-7015-12-22)
Supplement: Additional file 2 — Summary characteristics of reviewed comparison studies. [file 1741-7015-12-22-S2.pdf]

Additional file 2 - Summary characteristics of reviewed validation studies

No.	Setting	Period	Age group	Sample size	Causes of death	Interpretation method	Performance measures	Reference standard
1	Tanzania [28]	2000-2003	All	3123	All	PCVA	• Sensitivity<br>• Specificity<br>• Agreement of CSMF<br>• PPV	Hospital-based deaths
2	China [29]	2002	Adult	2102	All	PCVA	• Sensitivity<br>• Specificity<br>• Agreement of CSMF<br>• PPV	Hospital-based deaths
3	Thailand [30]	2006-2007	Adolescents and adults	2 558	All	PCVA	• Sensitivity<br>• PPV<br>• Agreement of CSMF	Hospital-based deaths
4	Mexico, Tanzania, India Philippines [31]	2005	All	12 542	All	PCVA	• Average of cause-specific chance-corrected concordance<br>• CSMFs accuracy<br>• Relationship between estimated CSMFs and true CSMFs	Hospital-based deaths
5	India [32]	1998-1999	Adults	48 357	All	PCVA	• Agreement of CSMF<br>• Sensitivity (only for cancer)	Death certificates
6	India [33]	1997-1998	Adults	31 788	All	PCVA	• Agreement of CSMF	Various types of records
7	Uganda	2006-	Adults	264	HIV –related death	PCVA	• Specificity	Clinically-

No.	Setting	Period	Age group	Sample size	Causes of death	Interpretation method	Performance measures	Reference standard
	34]	2008					• Agreement of CSMF<br>• PPV	confirmed HIV status
8	Uganda [35]	2008-2009	Children under 5	719	Malaria	PCVA	• Sensitivity<br>• Specificity<br>• PPV<br>• Agreement of CSMF	Hospital-based deaths
9	Kenya [36]	2002-2008	All	1 823	All	InterVA-3	• Kappa statistics<br>• Agreement of CSMF	PCVA
10	Burkina Faso [37]	1998-2007	All	5649	All	InterVA-3	• Agreement of CSMF	PCVA
11	Mexico Tanzania India Philippines [14]	2005	All	12 542	All	InterVA-3	• Chance-corrected concordance<br>• CSMFs accuracy<br>• Linear regression	Hospital-based deaths
12	South Africa [15]	1992-2005	All	1 000	All	InterVA	• Agreement of CSMF	InterVA-3
13	South Africa [38]	1992-2005	All	6,153	All	InterVA-3	• Agreement of CSMF	PCVA
14	Ethiopia [39]	2003	Adults	193	HIV/AIDS<br>HIV/AIDS+ TB	InterVA-3	• Sensitivity<br>• Specificity<br>• PPV<br>• Agreement of CSMF<br>• Kappa statistics<br>• ROC curves	Hospital-based deaths

No.	Setting	Period	Age group	Sample size	Causes of death	Interpretation method	Performance measures	Reference standard
15	Ethiopia [40]	2012	Adults	408	Pulmonary tuberculosis	InterVA-3	• Sensitivity<br>• Specificity<br>• Agreement of CSMF<br>• Kappa statistics<br>• Receiver operating characteristic curves	PCVA
16	Mexico<br>Tanzania<br>India<br>Philippines<br>[16]	2005	All	12 542	All	KL	• CSMF accuracy<br>• Linear regression	Hospital-based deaths
17	Mexico<br>Tanzania<br>India<br>Philippines<br>[12]	2005	All	12 542	All	Tariff	• Chance-corrected concordance<br>• CSMF accuracy<br>• Linear regression	Hospital-based deaths
18	Mexico<br>Tanzania<br>India<br>Philippines<br>[13]	2005	All	12 542	All	SSP	• Chance-corrected concordance<br>• CSMF accuracy<br>• Linear regression	Hospital-based deaths
19	Mexico<br>Tanzania<br>India<br>Philippines<br>[11]	2005	All	12 542	All	RF	• Chance-corrected concordance<br>• CSMF accuracy<br>• Linear regression	Hospital-based deaths
