# Supplementary material for: Comparison of physician-certified verbal autopsy with computer-coded verbal autopsy for cause of death assignment in hospitalized patients in low- and middle-income countries: systematic review
Source: BMC Med. 2014 Feb 4;12:22. doi: 10.1186/1741-7015-12-22 (PMC3912516; doi:10.1186/1741-7015-12-22)
Supplement: Additional file 3 — Chance-corrected concordance by cause for PCVA, Tariff, RF and SSP. [file 1741-7015-12-22-S3.doc]

Additional file 3- Chance-corrected concordance by cause for PCVA, Tariff, RF and SSP

|  |  | **PHMRC** | | |
| --- | --- | --- | --- | --- |
|  | **PCVA** | **Tariff** | **RF** | **SSP** |
| **Infections & parasitic diseases and maternal deaths** |  |  |  |  |
| Tuberculosis | 48.5 | 46.4 | 46.2 | 46.9 |
| HIV/AIDS | 58.8 | 53.75 | 57.7 | 63.6 |
| Diarrhoeal diseases | 43.7 | 35.6 | 42.3 | 28.4 |
| Malaria | 35.0 | 53.15 | 38.3 | 45.0 |
| Pneumonia | 23.7 | 16.57 | 27.1 | 19.5 |
| Other infections | 25.3 | 5.15 | 18.6 | 6.3 |
| **Maternal deaths** | 64.0 | 63.5 | 74.2 | 88.9 |
| **Neonatal conditions** |  |  |  |  |
| Prematurity and low birth weight | 42.8 | 23.02 | 27.6 | 21.7 |
| Birth asphyxia and birth trauma | 43.0 | 22.8 | 48.9 | 47.9 |
| Neonatal infections | 6.0 | 17.3 | 19.1 | 24.5 |
| **Noncommunicable diseases** |  |  |  |  |
| Digestive cancers | 35.4 | 30.8 | 27.8 | 36.7 |
| Respiratory cancers | 41.1 | 25.6 | 46.5 | 38.8 |
| Other cancers | 51.0 | 24.7 | 32.9 | 36.5 |
| Heart diseases | 57.4 | 32.6 | 37.4 | 50.4 |
| Stroke | 59.9 | 50.3 | 58.2 | 62.7 |
| Other CVD diseases | 9.5 | 28.0 | 29.5 | 9.2 |
| Chronic respiratory diseases | 42.6 | 47.2 | 42.6 | 48.6 |
| Cirrhosis of the liver | 46.2 | 37.3 | 59.4 | 65.2 |
| Other digestive diseases | 26.5 | 20.5 | 26.5 | 21.2 |
| Renal and other endocrine diseases | 22.3 | 19.1 | 33.0 | 11.7 |
| Other NCD | 35.2 | 10.7 | 21.6 | 3.4 |
| **Injuries** |  |  |  |  |
| Road traffic accidents | 85.2 | 66.2 | 84.8 | 78.6 |
| Other injuries | 61.1 | 60.7 | 57.3 | 56.8 |
| **All** | **41.9** | **34.4** | **41.6** | **39.7** |
|  |  |  |  |  |
